# Highways and outposts: economic development and health threats in the central Brazilian Amazon region

**DOI:** 10.1186/1476-072X-9-30

**Published:** 2010-06-17

**Authors:** Christovam Barcellos, Patrícia Feitosa, Giseli N Damacena, Marco A Andreazzi

**Affiliations:** 1 Health Information Research Department, Oswaldo Cruz Foundation, Rio de Janeiro, Brazil; 2 National School of Public Health, Oswaldo Cruz Foundation, Rio de Janeiro, Brazil; 3 Brazilian Census Bureau, Rio de Janeiro, Brazil

## Abstract

**Background:**

Economic development is often evoked as a driving force that has the capacity to improve the social and health conditions of remote areas. However, development projects produce uneven impacts on local communities, according to their different positions within society. This study examines the spatial distribution of three major health threats in the Brazilian Amazon region that may undergo changes through highway construction. Homicide mortality, AIDS incidence and malaria prevalence rates were calculated for 70 municipalities located within the areas of influence of the Cuiabá-Santarém highway (BR-163), i.e. in the western part of the state of Pará state and the northern part of Mato Grosso.

**Results:**

The municipalities were characterized using social and economic indicators such as gross domestic product (GDP), urban and indigenous populations, and recent migration. The municipalities' connections to the region's main transportation routes (BR-163 and Trans-Amazonian highways, along with the Amazon and Tapajós rivers) were identified by tagging the municipalities that have boundaries crossing these routes, using GIS overlay operations. Multiple regression was used to identify the major driving forces and constraints relating to the distribution of health threats. The main explanatory variables for higher malaria prevalence were: proximity to the Trans-Amazonian highway, high proportion of indigenous population and low proportion of migrants. High homicide rates were associated with high proportions of migrants, while connection to the Amazon River played a protective role. AIDS incidence was higher in municipalities with recent increases in GDP and high proportions of urban population.

**Conclusions:**

Highways induce social and environmental changes and play different roles in spreading and maintaining diseases and health threats. The most remote areas are still protected against violence but are vulnerable to malaria. Rapid economic and demographic growth increases the risk of AIDS transmission and violence. Highways connect secluded localities and may threaten local populations. This region has been undergoing rapid localized development booms, thus creating outposts of rapid and temporary migration, which may introduce health risks to remote areas.

## Background

Poverty is undoubtedly an important determinant of several diseases. On the other hand, economic development has been evoked as a process that has the capacity to break the disease and poverty cycles. Both social and geographic isolation have been held responsible for hindering social and economic development, especially in the Amazon region, where the distances and transportation difficulties are enormous.

However, it cannot be said that the Amazon region has remained on the sidelines of any development whatsoever. Some projects, with greater or lesser duration and reach, have taken place in the region and changed the way of life of some segments of the population, for instance in relation to soybean cultivation, ports and exportation corridors. Several projects such as the rubber production in Belterra, gold extraction in Itaituba and mining in Oriximiná, Almeirim and Monte Alegre have little scope for long-lasting development, despite being attractive to a large proportion of the population. This apparent contradiction has been explained by Bunker [1] as a characteristic of the extraction economy, which is short-lived and limited in scope due to its inability to create productive chains, or to retain wealth and the labor force. Accordingly, the profusion of development projects in the region has encouraged further mobility of the population and of capital, rather than the intended local development. These focal and volatile projects have been promoting "outposts" of development in relatively isolated areas, such as mining sites, attracting an extremely mobile and vulnerable population. Simultaneously, the region has experienced a more continuous change in society and ecology as a result of expansion of the economic frontier, from the southern part of the country, where industrial and financial activities are concentrated, to the Amazon region. In this respect, highways have played a role in inducing and supporting development projects.

In this context, highways are seen as drivers of development, both through facilitating the flow of goods between cities, and through making it possible to allocate land ownership for agriculture and extraction of natural resources. In fact, the highways that were previously constructed in the Amazon region, such as the Belém-Brasilia (BR-153) and Cuiabá-Rio Branco (BR-364) highways promoted an extraordinary transformation of the economy, embodied by changes in land use along their axes and surroundings [2,3]. However, in this and in other cases, development can be said to be "unequal and combined", as usually stated in the Marxist approach to political economy [4], affecting social groups differently depending on how they are positioned within society, overall. Accordingly, highways can be held responsible for the introduction of certain health problems and the reduction of others, in different places, at different times and among different social groups.

Few studies have reported on the influence of highways on health conditions, beyond the direct impact of traffic on accidents and air quality. Other health problems, such as the spread of communicable diseases, are likely to occur during and after the construction of a highway. Problems such as increasing land tensions, violence, malaria outbreaks and exposure to pesticides, among others, have become evident and have been associated with highway construction and paving [3,5-9]. Thus, the existence of a highway gives rise to health events, and these are mediated by changes in population habits, ecosystems, migration and land use [10-12]. The existing literature has often focused on a single health problem, such as a specific infectious disease, correlating its distribution and dynamics with highways. In the present study, we began our investigations from the standpoint that the presence of roads would be a source of risks, and then selected indicators for three major health threats based on the local population's concerns, as gathered during the early stages of the fieldwork, along with the technical criteria of availability, validity, responsiveness to changes, disaggregation capability, comparability and representativeness [13].

The AIDS epidemic has been spreading throughout Brazil, after its initial concentration in major metropolitan areas. In its current stage, the epidemic shows tendencies towards affecting the poor, women and rural populations [14], often following the main transportation routes [15,16]. Malaria is a disease that affects the entire Brazilian Amazon region. However, the intensity of its transmission depends on the characteristics of the local climate and vegetation, which affect the development of vectors (mosquitoes of the genus *Anopheles*), along with land use and living and working conditions. Together, these determine the level of the population's exposure to the vector [5,17]. Violence has been increasing throughout Brazil, most significantly in metropolitan regions and in frontier areas of economic expansion [18]. Some studies have highlighted social inequality as an important component for explaining violence [19]. Migration, disruption of family and social structures, absence of governmental support and impoverishment are socioeconomic factors that affect local social dynamics and lead to violence.

In this study, AIDS, malaria and violence were chosen as health-sensitive indicators, in order to examine the differential impact of highways and other connections on sociodemographic structure, changes in regional economic dynamics and health conditions.

**Figure 1:**
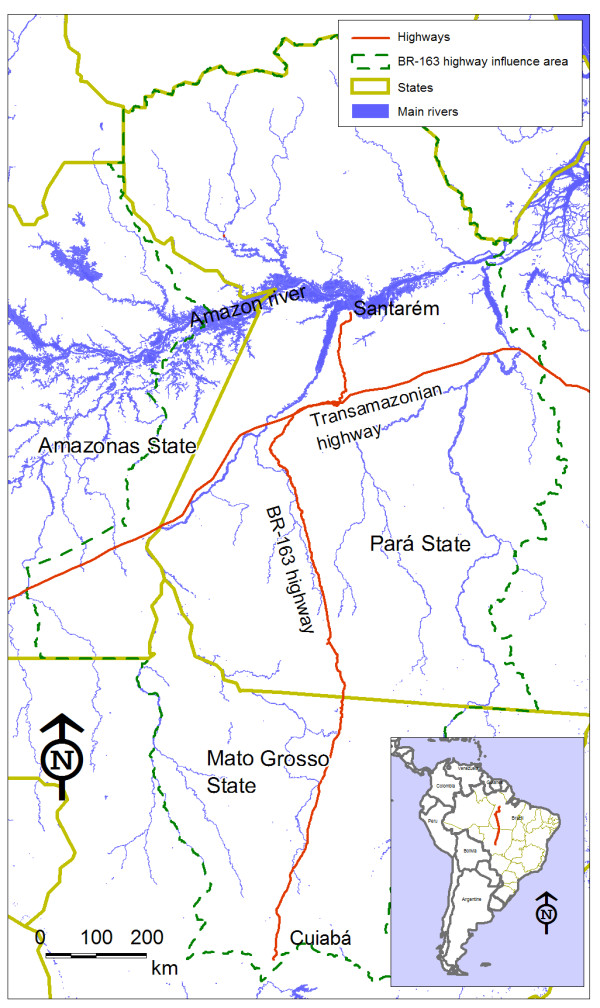
**Configuration of study area: main rivers, state limits and highways in the area of influence of the BR-163 highway**.

Figure 1 shows the context within which the BR-163 highway is situated. The Amazonian section of the BR-163 highway was constructed in the early 1970s with the aim of stimulating agricultural colonization and occupation of the "demographic vacuum" in the northern part of the state of Mato Grosso and the western part of the state of Pará, as argued by strategists under the influence of the Brazilian military regime (1964-1984). The current condition of the highway restricts the flow of vehicles, especially during the rainy season (December to June), during which mud accumulates on the road surface and small bridges may crumble. Consolidation of the BR-163 highway, by paving the stretch that passes through the state of Pará, has been demanded by the region's population, and by economic interest groups linked to soybean cultivation [20]. This undertaking has the potential to affect an area of 974,000 km^2^. According to the sustainable development plan for the BR-163 area of influence [21], it is expected to accelerate unprecedented waves of migration, illegal appropriation and occupation of public lands, land concentration, deforestation, rising crime and insecurity of health conditions. The initial estimates predict deforestation of over 180,000 km^2 ^of Amazon forest in a few years' time. An additional likely consequence of consolidation of this road is the endemic malaria in the region will be worsened, and there may even be outbreaks of sexually transmitted infections (STIs) and AIDS among migrants, as well as increases in violence and accidents. The increased population flow predicted for the region will, in combination with the diversification of risk factors and diseases that the region will be subjected to, create demand pressure on the health service network.

Given the prospect that the highway will be consolidated over the coming years, this study had the aim of examining the role of transportation routes in the dynamics of health problems, through measuring associations between the presence of highways and waterways and health conditions. At the same time, a baseline level was outlined to make it possible to monitor the impact of the BR-163 highway throughout its area of influence. The incidence of malaria, AIDS and homicide mortality were used as marker events associated with the likely changes occurring in this region.

The influence area of the highway was defined in accordance with the "Sustainable BR-163" plan, such that it comprised a total of 70 municipalities: 6 in the southeastern part of the state of Amazonas, 28 in the southern part of the state of Pará, and 36 in the northern part of the state of Mato Grosso [21]. The area of Mato Grosso has, over recent decades, undergone significant changes because of the introduction of soybean cultivation, livestock raising and deforestation. This area has also experienced rapid population growth resulting from the migration of small- business owners and workers from southern and northeastern Brazil. At the same time, the state of Pará maintains traditional farming methods such as cattle husbandry in wetland areas, artisanal rubber production and harvesting of Brazil nuts. This area also presents important bauxite mining activities in Oriximiná, Almeirim and Monte Alegre, and gold mining in Itaituba. Therefore, the boundary between the states of Mato Grosso and Pará corresponds roughly to the frontier of agricultural expansion, largely due to obstacles relating to crop production and flow along the length of the BR-163 highway that runs through the state of Pará.

The other important routes in the region are the Trans-Amazonian highway (BR-230) and the Tapajós and Amazon rivers. Rivers are a widely-used transportation alternative in the Amazon River region, because of the poor maintenance of highways and the way in which, historically, towns and villages grew up along major rivers [22].

However, the region's economic and population dynamics are not solely a consequence of highway and river networks. The presence of mining operations, either as formal or informal ("garimpo") activity, in remote areas of the region has shown that, in several cases, transportation alternatives were created only after economic activities became established. Such activities have great potential to attract a labor force, thereby rapidly changing the demographic structure of small municipalities.

## Methods

### Indicators and statistical analysis

In this study, the following indicators were used to analyze health conditions and factors relating to transportation and to the economy in municipalities within the area of influence of highway BR-163:

• Mean homicide mortality rate from 2000 to 2005 according to the city of residence, calculated as the total number of deaths classified in ICD-10 between E960-E969 (homicides) and E985-E986 (injury by firearm or perforating weapon) divided by the total population, multiplied by 100,000.

• Mean annual parasitic index (API) from 2003 to 2005, calculated as the total number of positive malaria tests, divided by the total population estimated in each municipality, multiplied by 1,000.

• Mean AIDS incidence rate from 2000 to 2005, calculated as the total number of reported cases of AIDS divided by the total population estimated in each municipality, multiplied by 100,000.

• Total population in the municipality in 2000, according to the demographic census.

• Geometric growth rate of the population in the municipalities in question between censuses (1991 to 2000), using data obtained from the demographic census.

• Proportion of the population classified as urban, obtained from the 2000 demographic census.

• Proportion of indigenous population, using data obtained from the 2000 demographic census.

• Rates of recent migration (less than two years) and old migration (over 10 years), obtained from demographic censuses (1996-2000) and from data made available by the Brazilian Institute of Applied Economic Research (IPEA) [23].

• Gross domestic product (GDP) for 2005, in R$, calculated by the Brazilian Institute for Geography and Statistics (IBGE) and by IPEA [23].

•Growth rate of the gross domestic product (GDP) between 2000 and 2005, calculated by IBGE and by IPEA [23].

• Cost of transportation (in US$) to the nearest state capital, in 2000, calculated by IBGE and by IPEA [23].

• BR-163 highway: a dichotomous variable obtained by overlaying municipality limits and the axis of highway BR-163. If the highway crossed the limits of the municipality, it took a value of 1; otherwise, the variable took a value of 0.

• Amazon River: a dichotomous variable obtained by overlaying the municipality limits and the margins of the Amazon River. If the river crossed the municipality limits, the variable took a value of 1; otherwise, the variable took a value of 0.

• Trans-Amazonian highway: dichotomous variable obtained by overlaying the limits of the municipality and the axis of the Trans-Amazonian highway. If the highway cut the limits of the municipality, this took a value of 1; otherwise, the variable took a value of 0.

• State of MT: dichotomous variable indicating whether the municipality belongs to the state of Mato Grosso.

It was possible to build up a historical series of health indicators, from 1980 to 2005, except for malaria incidence, for which data was recovered from 2003 to 2005.

The indicators were calculated on spreadsheets, then verified and associated to a digital database of municipalities. Other data layers were added to build a geographic information system (GIS), using Mapinfo software: major rivers, major highways and towns. The GIS also enabled construction of thematic maps that were analyzed to study the spatial distribution of selected indicators. The indicators of transportation routes (BR-163, Trans-Amazonian Highway and Amazon River) were calculated using GIS Boolean expressions that combined the municipality and highway layers, with incorporation into the matrix of indicators. The municipalities that were crossed by highways or rivers were assigned a dichotomous variable that identified the potential connection to regional production and consumption centers. The resultant matrix was then exported to the SPSS statistical software, version 13.0, in which univariate analyses (normality tests) and multiple linear regression analyses were performed. Health indicators (AIDS, malaria and homicide) were considered to be dependent variables in the regression analysis, while socioeconomic indicators and transportation lines (highways and major rivers) were taken to be independent variables. The final variables in the models were selected by means of the backward procedure, starting from the initial list of variables and sequentially removing the variable with the smallest partial correlation with the dependent variable. The final set of independent variables was considered optimal when the model reached the maximum adjusted R^2 ^(minimum standard error of estimate), leaving four independent variables. Some of the variables contained in the final list were not significantly associated with the health outcomes when considered separately, although they contribute to the overall model fit.

## Results

### AIDS, violence and malaria distribution

**Figure 2:**
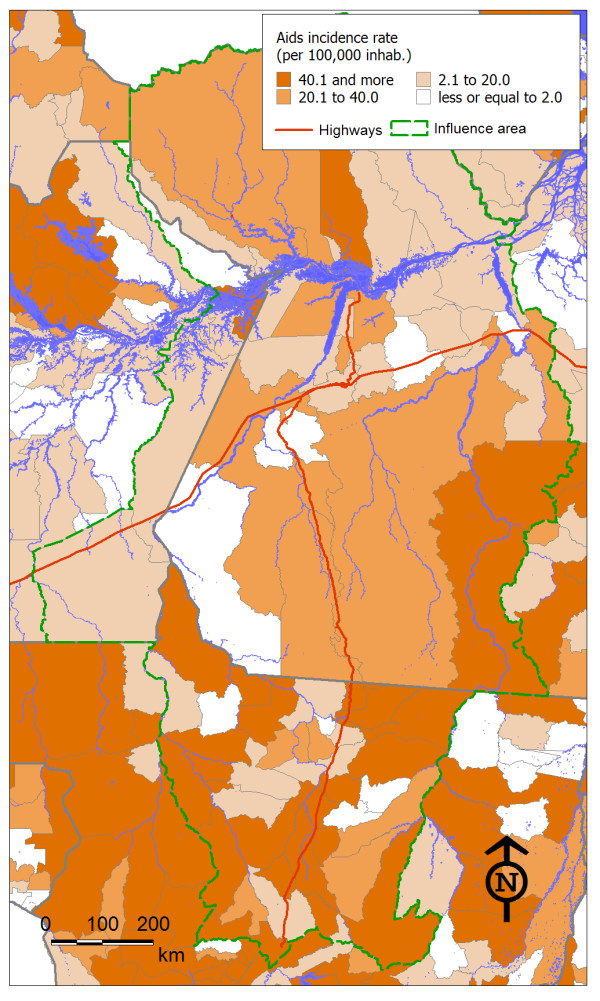
**Spatial distribution of AIDS incidence rate in municipalities in the area of influence of the BR-163 highway**.

**Figure 3:**
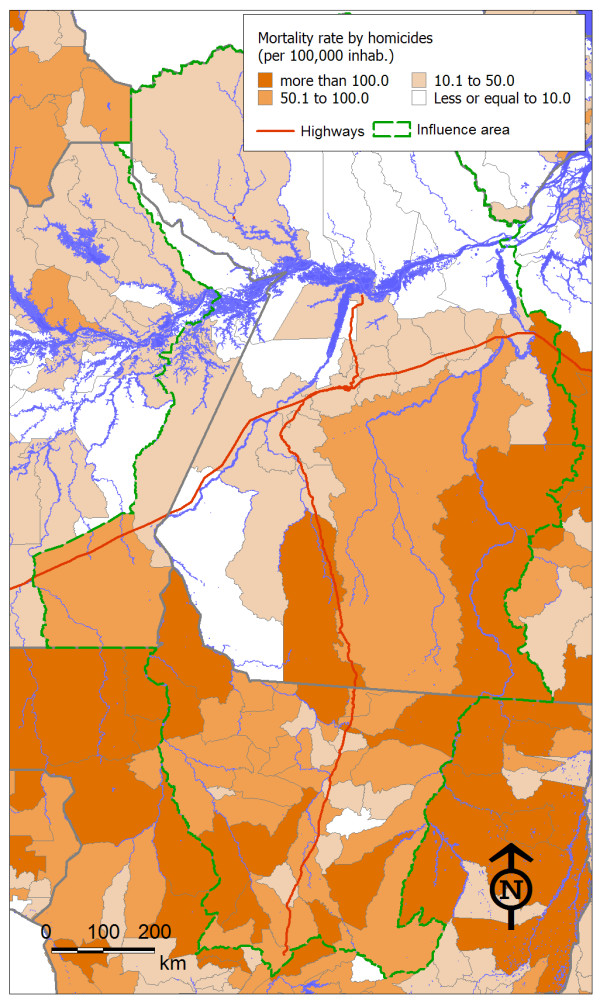
**Spatial distribution of homicide mortality rate in municipalities in the area of influence of the BR-163 highway**.

**Figure 4:**
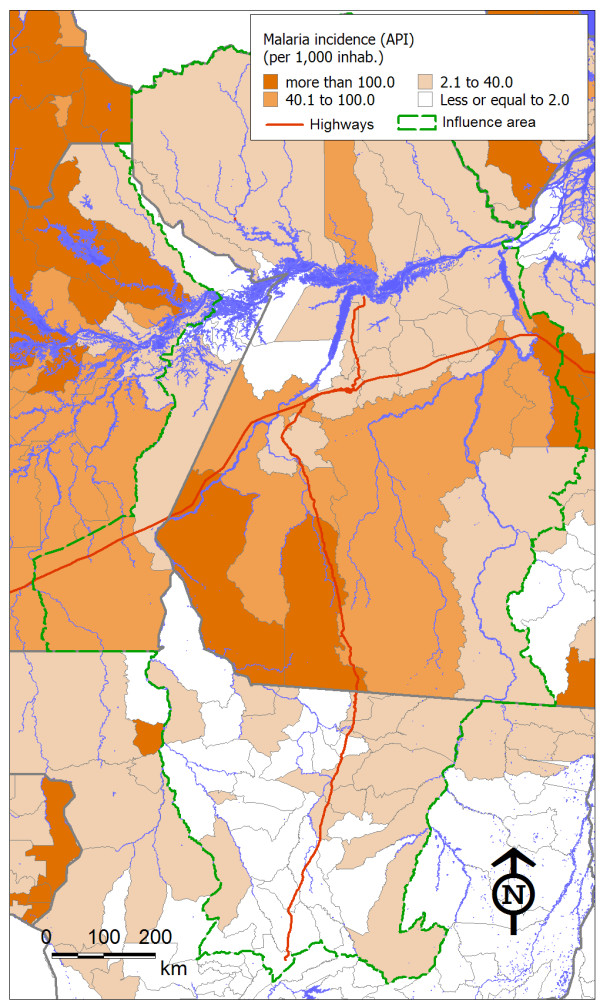
**Spatial distribution of malaria incidence in municipalities in the area of influence of the BR-163 highway**.

As expected, the indicator values showed great variation among the municipalities in the region. Figures 2, 3 and 4 show the spatial distribution of the three health threats studied, which were considered to be sensitive to changes in the economy and the local population dynamics as an outcome from highway construction. The maps offered a view of how these diseases had different spatial distributions, which probably reflected their different social and environmental determinants.

The state of Mato Grosso had the largest number of municipalities with AIDS incidence rates higher than 40 per 100,000 (Figure 2). Such rates are considered high in comparison with the nationwide value of 17.7 per 100,000 [24]. Outside of this state, the rates were generally lower, except for the municipalities of Alenquer and São Feliz do Xingu (both in Pará state) and Parintins (in Amazonas state).

Likewise, for deaths due to homicide, the state of Mato Grosso showed higher rates (Figure 3) than seen in the states of Amazonas and Pará. However, in Mato Grosso, the homicide rates were lower in municipalities crossed by BR-163. Higher mortality rates predominated in the eastern portion of the state of Pará. The municipalities north of the Amazon River generally had lower mortality rates due to homicide.

The map (Figure 4) shows that malaria had a higher impact in the state of Pará than in Mato Grosso, unlike the other two indicators. The highest incidence rates in Mato Grosso were in cities away from the BR-163 highway, while in Pará the incidence was higher in municipalities in the southern portion of the state, such as Jacareacanga and Novo Progresso.

### Model results

Table 1 shows the main variables in the multivariate regression models for health indicators.

**Table 1:** Main explanatory variables for the spatial distribution models for AIDS, homicides and malaria. Beta coefficient and confidence intervals of variables in linear regression models

		State of MT	BR-163 highway	Amazon River	Transamazonian highway	GDP growth	Proportion of urban population	Proportion of indigenous population	Proportion of migrants (10 years)
AIDS	Beta (Confidence interval)	10.2 (-3,3 - 23,7)	-8.9 (-25,9 - 8,2)			1.4 (-2,2 - 5.0)	0.9* (0.5 - 1.3)		
Homicides	Beta (Confidence interval).	22.3* (3.5 - 41.1)	-13.5 (-35.8 - 8.8)	-43.7* (-72.4 - -15.0)					-1.3* (-2.2 - -0.4)
Malaria	Beta (Confidence interval)	-10.5 (-26.8 - 5.8)			56.9* (28.6 - 85.2)			2.3* (0.1 - 4.4)	-0.8 (-1.6 - 0.1)

* significant (p < 0.05)

The main variables explaining the incidence of AIDS in the region's municipalities were: belonging to the state of Mato Grosso, not being crossed by the BR-163 highway, high GDP growth over recent years and high proportion of urban population. The model was highly significant (p = 0.002) and explained 37% of the AIDS incidence variation among the municipalities (R = 0.61).

In the case of homicide, the fact that a municipality belonged to the state of Mato Grosso contributed towards a higher mortality rate. On the other hand, proximity to the BR-163 highway and the Amazon River, along with a high proportion of migrant population over the last 10 years, acted as a protective contextual factor. The model explained 44% of the homicide rate variation among municipalities (R = 0.66), which was considered highly significant (p < 0.001).

Regarding malaria, the municipalities with the highest incidence rates were crossed by the Trans-Amazonian highway, had a high proportion of indigenous population, belonged to the states of Pará or Amazonas and had a low proportion of migrant population over the last 10 years. The model was highly significant (p < 0.001) and explained 33% of the homicide rate variation among municipalities (R = 0.57), which was considered highly significant (p < 0.001).

We chose to use models that reduced multicollinearity among the variables to a minimum, which left us with four variables. The variables of total population, population growth rate, gross domestic product value and the cost of transportation to the state capital were excluded because of their insignificant contribution to the overall model fit.

**Figure 5:**
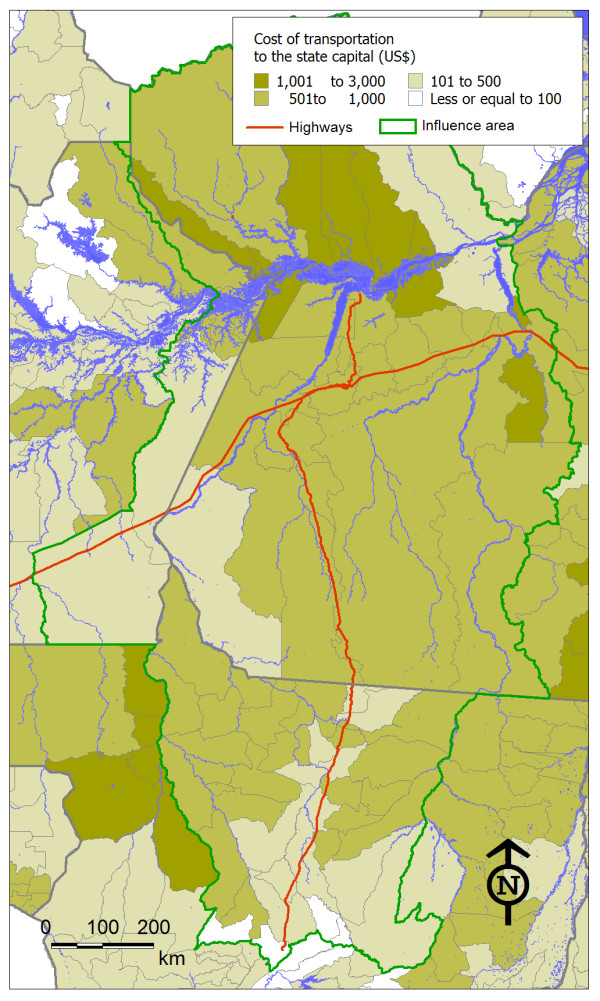
**Cost of transportation to the state capital (in US$) in the municipalities within the area of influence of the BR-163 highway**.

However, it is worth noting the associations that existed between the cost of transportation and other indicators employed in this study. This variable showed significant inverse correlations with the AIDS incidence rate (p = 0.04), homicide mortality rate (p = 0.002), proportion of urban population (p = 0.003), GDP value (p = 0.03) and proximity to BR-163 highway (p = 0.01). The cost of transportation had a significant direct correlation with the proximity of the Amazon River (p < 0.001). Figure 5 illustrates the cost of transportation to state capitals of the municipalities within the area of influence of the BR-163 highway.

The costs were generally higher in the state of Pará and lower in the state of Mato Grosso. In the latter state, the costs were lower in municipalities crossed by BR-163, while in Pará, there was a high cost of transportation to the capital (Belém), mainly along the Amazon River. Ironically, the presence of highways in Pará state, i.e. the unpaved stretch of the road, did not significantly alter the costs of transportation, which may be a consequence of the bad road conditions, particularly during the wet season. As mentioned above, the present condition of the northern stretch of the BR-163 highway restrains the traffic of goods and people.

## Discussion

All three multiple regression models showed high correlation levels (all with p < 0.01), thus indicating the adequacy of the variables used to explain the spatial distributions of AIDS, malaria and homicides. The highways (BR-163 and Trans-Amazonian) or the isolated development outposts (represented by the indicators of GDP increase and proportion of the population that had migrated there for less than 10 years), considerably altered disease distribution in this area of the Brazilian Amazon region.

The BR-163 highway has already produced significant changes in northern Mato Grosso. This has primarily occurred through cutting freight costs, but it has also changed land use through a series of events that began with disputes over land ownership, continued with deforestation ("timber market"), then with livestock rearing (creating a "cattle market") and finally with the introduction of crops of higher added value, such as soybeans [25]. In the state of Pará, these changes are still taking place slowly and only in restricted areas. The precarious highway maintenance is a major barrier to economic development, i.e. a barrier to consolidation of productive chains in the area. The evaluation of transportation costs showed that the presence of the BR-163 highway in the state of Pará has not reduced the costs. Along the Amazon River, where fluvial transportation is predominant, these costs are very high, and among the highest recorded in the country. In many cases, these high costs act as an obstacle to the installation of large businesses, but they are also a barrier to the riverside population, through reducing the opportunities for marketing their artisanal products and hampering transportation as a whole [26]. On the other hand, these same obstacles may be responsible for protecting the local population from the well-known problems associated with "development". In this study, we showed that transportation costs were inversely associated with AIDS incidence and homicide mortality rates.

Mainly since the 1990s, the AIDS epidemic has been moving towards the frontiers of economic expansion. Migration, prostitution networks and disruption of social structures exacerbate AIDS transmission in pioneer areas [27,28]. In this study, there were high AIDS incidence rates in most of the northern portion of Mato Grosso, and the same has happened in municipalities undergoing rapid development in the states of Pará (Alenquer and São Felix do Xingú) and Amazonas (Parintins). These municipalities also present higher proportions of AIDS cases resulting from injected drug use. The role of injected drug users (IDUs) in the spread of HIV has been portrayed previously in other regions of Brazil [29]. The existence of drug-dealing locations, primarily cocaine coming from Peru and Colombia, may facilitate drug use. These corridors of drug distribution coincide with those that existed in the colonial past throughout port cities in the Brazilian Amazon region [30]. Small and illegal or informal mining ("garimpo") activities also act as outposts of HIV transmission, although apparently isolated from large urban centers [31].

The average rates of violence in the area of influence of the BR-163 highway within the state of Pará were higher. However, as shown on the homicide rate map (Figure 3), the state of Mato Grosso contains a larger number of municipalities with high values. This dual condition indicates that violence in the state of Pará is much more concentrated in few municipalities, such as São Felix do Xingu, Altamira and Novo Progresso. Thus, transportation routes such as the BR-163 highway (in its present poor condition) and the Amazon River may act as barriers to this type of violence. The sequence of maps of homicide rate [see Additional file 1] shows the spread of violence along the area of influence of the BR-163 highway.

Long term migration (10 years of residence) showed an inverse association with homicide mortality rates (r = -0.445). It is interesting to observe that homicide mortality was higher in the municipalities with large proportions of recent migrants (2 years of residence) (r = 0.415), with a direct association. Municipalities that were subjected to earlier migration waves tended to have lower homicide rates, while municipalities with more recent migration tended to have higher homicide rates. This trend shift relating to violence, as a result of a longer migration period, prompted us to consider the role of economic cycles in producing social stress. The first events of these occupation cycles are marked by land value increases and a series of conflicts between settlers, "grileiros" (land-grabbers) and the traditional local population for land ownership, often obtained through illegal titles, corruption and violence. Timber extraction also often occurs illegally, thereby promoting a chain of illicit processing and marketing [32]. All these activities are potentially drivers of interpersonal conflicts and stimulate the formation of illegal groups. In municipalities where migrants have been settled for longer periods, economic activities are more stable, such as traditional agriculture and fishing. In these places, transportation costs are higher (r = 0.252) and there is less urban population (r = - 0.329). In the words of Milton Santos [33], these places are marked by "slow time" and are reluctant to adapt to the speed of change. This isolation probably protects local people against the violent effects of economic development.

The areas of greatest intensity of malaria transmission were mainly located in the southern part of the state of Pará and along the Trans-Amazonian highway. This area is situated along the occupation frontier, where deforestation and forest fires are most intense. The deforestation practices today are repeating the same timber felling pattern as seen in other areas of the Brazilian Amazon region and in the state of Rondônia during the 1980s, which was responsible for major malaria outbreaks in the Amazon region [5]. Areas that have been occupied and cleared for longer periods tend to show decreases the prevalence of malaria, as seen in the northern portion of Mato Grosso since the beginning of the current decade [34].

It is worth noting that the main highways in the region presented different effects on the health outcomes studied here, as represented by the model coefficients in Table 1. The Trans-Amazonian highway increased the risk of malaria transmission in the municipalities located along its axis, while the BR-163 highway had no impact on malaria incidence. On the other hand, municipalities crossed by the BR-163 highway presented slightly lower homicide rates than did the unconnected municipalities. The uneven effect of highways on health conditions can be interpreted in terms of their underlying economic development project. The first occupation wave in the central Amazon region originated from rubber tree exploitation during the final decades of the nineteenth century. The population was dispersed along the main Amazonian rivers and wealth was concentrated in Manaus and Belém, the capitals of the states of Amazonas and Pará, respectively. Construction of the Trans-Amazonian highway (BR-230) during the 1970s promoted large-scale migration to the region following the distribution of land parcels along the highway. This started a long-lasting process of competition for land and natural resources between migrants and the local population [35]. The third wave of central Amazon occupation is embodied by the project to pave the BR-163 highway. The southern portion of the highway, located in the state of Mato Grosso was rapidly converted for crop cultivation, thereby accelerating migration and land clearance. However, in the northern area along the highway, in the state of Pará, large changes in land use are still hampered by legal and climatic constraints [36].

## Conclusions

This ecological study sought to identify the role of transportation networks as determinants of health problems. First, health indicators sensitive to economic and demographic dynamics were selected. We then used contextual indicators that reflected the transportation network in the area of influence of the BR-163 highway. This type of study has no validity for identifying the causes of health problems at the individual level. Its importance lies in its ability to recognize contextual and socioeconomic factors, allow diagnostic mapping of a situation and establish scenarios regarding regional development. The unit of analysis adopted was the municipality, which constitutes an important level for the development of public policies aimed at protecting the population's health. The strategy of this study is in agreement with the proposed policies for reducing the negative impact of road construction and paving in the Amazon region, which are territorially based. For instance, recent laws have created parks and protected areas around the BR-163 highway, thus forming a buffer of restricted land use in order to protect the region from the consequences of the expected wave of migration, deforestation and introduction of new and massive agriculture practices [37]. The federal government has launched the "Territories of Citizenship" program (Programa dos Territórios de Cidadania) in order to expand and integrate social services such as energy, water supply and sewerage systems, social assistance, education and basic health services, among others [38]. Three of these territories cover areas with low ratings on the Human Development Index (HDI) in the BR-163 area of influence.

The area of influence of the BR-163 highway presents a wide range of epidemiological and socioeconomic conditions, as a result of diverse and simultaneous processes. There is an association between these two components, which is largely mediated by highway and transportation conditions in the region. Highways induce profound changes in the social and environmental surroundings [32], and have different roles in maintaining or spreading diseases and health threats. The more remote areas are protected against violence, but are vulnerable to malaria. Rapid economic growth and population increases tend to enhance vulnerability to AIDS and violence. This diversity of health situations is governed by differences in timing and pace that are imposed by local connections, i.e. the networks between the municipalities.

Our work has shown that there is a strong link between the regional development model and the population's health profile. A great diversity of situations is evident, as well as their relationship to macrostructural factors, in which transportation conditions play an inductive role.

For decades, the Brazilian Amazon region has experienced booms of rapid and self-contained development around mining and timber extraction activities. More than enclaves, these businesses have acted as magnets for labor, thereby shaping local economic, political and social life [39]. According to Bertha Becker [40], the Amazon region is undergoing structural changes led by connectivity, industrialization, urbanization and organization of civil society and socioenvironmental networks. The whole region will not necessarily suffer the same succession of occupation, starting with deforestation, and moving on to livestock rearing and soybean agriculture, as has been observed in areas of the state of Mato Grosso. The observations depicted from this work do not imply a linear and sequential perspective of development in the region. On the contrary, they represent a wide diversity of health situations and their connection with the economic and sociopolitical structures.

## List of abbreviations

AIDS: Acquired Immune Deficiency Syndrome; API: Annual Parasitic Index for malaria; GDP: Gross Domestic Product; GIS: Geographic Information System; HDI: Human Development Index; IBGE: Brazilian Institute for Geography and Statistics; IDU: Injecting Drug Use; IPEA: Institute of Applied Economic Research; STIs: Sexually Transmitted Infections

## Competing interests

The authors declare that they have no competing interests.

## Authors' contributions

CB and MAA conceived the study, while GND conducted the statistical analysis. CB and PF wrote the manuscript. All authors approved the final manuscript.

## Supplementary Material

Additional file 1 **Spread of violence along the area of influence of the  BR-163 highway.** Dynamic map showing the evolution of homicide rates  (1980-2005) in the central Brazilian Amazon region. The homicide events were geocoded according to the respective city (center of the municipality) and the number of homicides was divided by the total population of the municipality using a factor of 100,000 inhabitants. A grid of .5 × 0.5 degrees was created and the homicide rate was assigned to the respective cell where the city is located.
The movie is in WMV format, which can be viewed using Media Player, Quick Time Player or similar software. Click here for file
